# Qualitative analysis of mental health conversational agents messages about autism spectrum disorder: a call for action

**DOI:** 10.3389/fdgth.2023.1251016

**Published:** 2023-12-05

**Authors:** S. Aghakhani, N. Carre, K. Mostovoy, R. Shafer, K. Baeza-Hernandez, G. Entenberg, A. Testerman, E. L. Bunge

**Affiliations:** ^1^ Department of Psychology, Palo Alto University, Palo Alto, CA, United States; ^2^ Fundacion ETCI, Buenos Aires, Argentina

**Keywords:** autism spectrum disorder, chatbots, digital interventions, digital mental health, technology

## Abstract

**Background:**

Conversational agents (CA's) have shown promise in increasing accessibility to mental health resources. This study aimed to identify common themes of messages sent to a mental health CA (Wysa) related to ASD by general users and users that identify as having ASD.

**Methods:**

This study utilized retrospective data. Two thematic analyses were conducted, one focusing on user messages including the keywords (e.g., ASD, autism, Asperger), and the second one with messages from users who self-identified as having ASD.

**Results:**

For the sample of general users, the most frequent themes were “others having ASD,” “ASD diagnosis,” and “seeking help.” For the users that self-identified as having ASD (*n* = 277), the most frequent themes were “ASD diagnosis or symptoms,” “negative reaction from others,” and “positive comments.” There were 3,725 emotion words mentioned by users who self-identified as having ASD. The majority had negative valence (80.3%), and few were positive (14.8%) or ambivalent (4.9%).

**Conclusion:**

Users shared their experiences and emotions surrounding ASD with a mental health CA. Users asked about the ASD diagnosis, sought help, and reported negative reactions from others. CA's have the potential to become a source of support for those interested in ASD and/or identify as having ASD.

## Introduction

Autism spectrum disorder (ASD) is highly prevalent, and it is estimated that one in fifty-four individuals in the United States of America [USA (1);] and 0.6% globally (2) have ASD. Common characteristics of ASD include deficits in social communication, restricted repetitive behaviors, and ASD is often associated with social, academic, and occupational impairments (3, 4). Individuals with ASD* face challenges related to accessing treatment, such as stigma and long waitlists (5, 6). Additionally, the aggregate cost of supporting people with ASD in the USA is estimated at $196 billion for adults (7). Furthermore, adults with ASD experience significant difficulties finding providers willing to treat them or who have knowledge of ASD (8), and many report feeling misunderstood by professionals (5).

Technology has the potential to increase access to resources and information, provide support in daily activities, promote independence, and assist with occupational skills for individuals with ASD (9). In recent years, there has been an increase in studies on digital tools for ASD (10). Some examples include social skills training through virtual reality [VR; (11)], cognitive and face-processing training through serious games (12), and augmentative and alternative communication (AAC) through tablets used as speech-generating devices (13). Although VR, serious games, or AAC tools can facilitate the learning process, their ability to promote interaction is limited, and they often do not mimic conversations with humans.

Conversational agent (CA or Chatbot) interventions have been increasingly adopted over the last decade as a viable resource for mental health care (14). CA's can offer context-specific and continuous accessible support, and studies on chatbots for mental health have shown promising clinical outcomes thus far (15, 16). In a meta-analysis on chatbot-delivered psychotherapy in adults, significant improvements were found in depressive symptoms (17). Other studies have shown promising outcomes in the reduction of attention-deficit/hyperactivity symptoms for adults (18), anxiety symptoms in college students (19, 20), and chronic pain (21). Additionally, chatbot utilization has been associated with higher retention rates compared to other digital interventions (17, 21), increased service utilization for individuals with eating disorders (22), and has assisted with teaching parenting skills (23).

The interactive and conversational format of chatbots led to a series of interesting findings showing that adults can form a positive relationship with a chatbot (24–26). Dosovitsky and Bunge (26) reported that users perceived the CA as safe, non-judgmental, caring, and open to listening. In the study by Beatty et al. (24), participants reported feeling gratitude and appreciation toward a therapeutic chatbot and reported levels of therapeutic alliance similar to in-person studies. The users in Darcy et al. (25) study also formed a therapeutic alliance with a CA within a few days, at levels comparable to in-person studies, and remained stable after eight weeks. These studies have important clinical implications given that if individuals with ASD can form a relationship with a chatbot, this may be an avenue for additional support and user-friendly interventions for this underserved population. However, there is a limited number of studies that have focused on the use of chatbots by individuals with ASD.

A review of a mental health CA (27) found ten chatbot studies focusing on ASD. Most of these studies aimed to improve social skills (28–30) or job interviewing skills (31), and one study focused on ASD assessment (32). Razavi et al. (30) and Ali et al. (28) tested Live Interactive Social Skills Assistant (LISSA), which was created to provide real-time feedback to adolescents with ASD about their nonverbal behaviors and to help them practice conversational skills. In both studies, participants provided mixed reviews, and the samples were small; five participants in Razavi et al. (30) and nine in Ali et al. (28). Lahiri et al. (29) designed a VR-based adaptive response technology to assist children with ASD with social interactions and found that social communication skills could be improved by utilizing this technology (29). Smith et al. (31) tested a virtual reality job interview training (VR-JIT), an interactive role-play simulation designed to help individuals with ASD improve their performance in a job interview. A preliminary study showed that VR-JIT effectively increased users' performance scores (31). Mujeeb et al. (32) created an ASD assessment chatbot and showed that, when compared to human psychologists, Aquabot had 88% accuracy in diagnosing ASD.

While studies on CA's have shown promising outcomes, they still present several limitations. Chatbots have a limited ability in understanding the nuances of users' experiences (33). Many studies reported users feeling annoyed by the lack of understanding or technical problems (26, 34, 35), as well as the unnatural flow of the conversation (18). Chatbot studies for ASD present similar limitations. The sample sizes of the chatbot studies for ASD are small, mostly case studies, and focused on children with ASD rather than adults (36, 37). Thus far, no studies have assessed how individuals with ASD use a chatbot to address issues related to mental health.

With a high prevalence of ASD (1), access to affordable and accessible interventions is needed. CA's have shown promise in increasing the accessibility and affordability of mental health resources. However, there are no studies showing what users share about ASD with a mental health chatbot. More specifically, this study will aim to (1) identify common themes of conversations related to ASD of all users, with and without ASD, interacting with a mental health CA; (2) common themes of conversations from individuals that self-identify as having ASD interacting with a mental health CA; (3) identify the most frequent emotion words individuals with ASD utilize when interacting with the CA; and (4) identify the most frequent life challenges individuals with ASD select from a CA for mental health.

## Method

The current study utilized retrospective data on users of a mental health chatbot and was determined non-human subject research by the Institutional Review Board (FWA00010885).

### Participants

The sample of users was pulled from a mental health CA, Wysa, and divided into two groups. A total of 1,397 messages corresponding to 908 unique users were screened. Wysa indicates that users must be over 18 years of age; however, users between 13 and 18 years of age are asked to read the Terms of Service and Privacy Policy along with their parents or legal guardian.

The first sample consisted of general users that mentioned ASD in their conversations. The sample of general users might have included users with and without ASD, but it was not possible to determine how they identified themselves.

**Figure 1:**
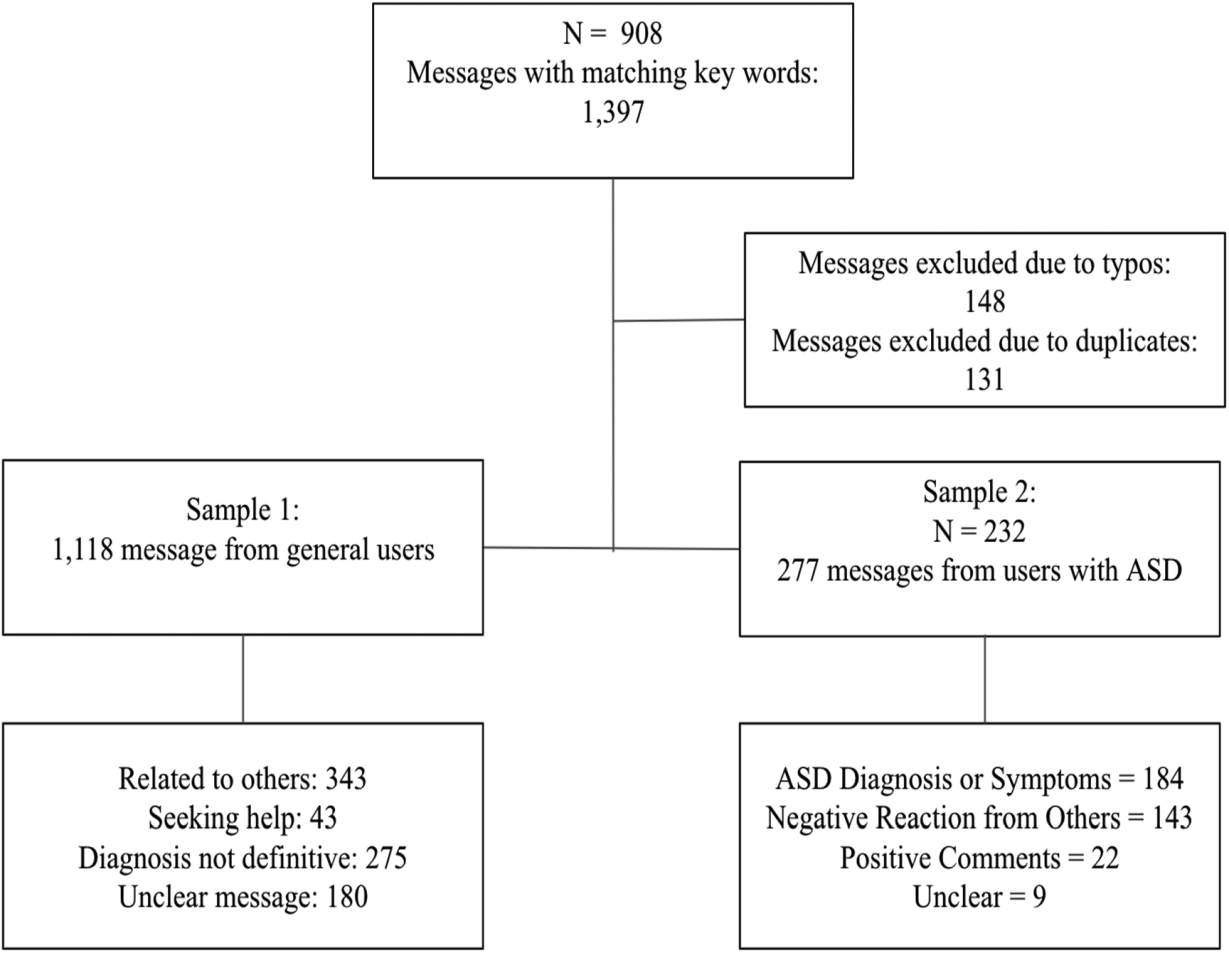
Screening process of messages mentioning words related to autism spectrum disorder.

The second sample consisted of users who self-identified as having ASD and were included if their messages met the following criteria: (1) the user reports ASD in first-person (e.g., I have autism); and (2) the report is definitive (e.g., my autism). Participants were excluded if the message was about someone else experiencing ASD (e.g., my partner has autism); if they expressed doubts about having ASD (e.g., I think I have ASD); if the message was a question about ASD (e.g., Do you think I have ASD?); or if the message was ambiguous [e.g., “meltdown autism” (SIC)]. The sample for this study was collected from regular Wysa users, an anonymous nonclinical population. The stringent exclusion criteria resulted in 277 messages from 232 unique users (See Figure 1).

## Materials

### Wysa

Wysa is an anonymous artificial intelligence (AI) conversational agent publicly available to a nonclinical population as an app on the Android and iOS app stores. It interacts with the users through a free-text conversational interface as users downloaded the app voluntarily after providing consent for the Terms of Service and Privacy Policy. Wysa prioritizes security, safety, and privacy, as no identifiable information is collected throughout app usage (38). For privacy reasons, the authors had access to limited deidentified conversational messages and extracted identified keywords. Users are informed about their rights to exclude data shared with the app for research purposes. Wysa recommends resources based on evidence-based therapies, such as cognitive behavioral therapy (CBT) and mindfulness (39, 40). Wysa aims to build mental resilience and promote mental well-being (16). The techniques and self-care strategies recommended include targeting areas that increase joy, provide opportunities to reflect positively, reframe cognitive distortions, increase gratitude, and find acceptance (41–43). Wysa's services are free and always available. In addition, there is an option to access a human coach and some additional tools for a fee (16).

## Data extraction

### Messages

Messages scanned were sent between June 1st, 2021, and July 11th, 2022. Messages were screened using a set of keywords: autistic, autism, autism spectrum, autism spectrum disorder, ASD, autistic disorder, on the spectrum, Asperger, Aspergers, Asperger syndrome, pervasive developmental disorder, PDD, Rett, and Retts. Two samples were generated, one with general users who mentioned keywords related to ASD and another with users who self-identified as having ASD. Two independent researchers reviewed messages, and disagreements were solved by consensus.

### Emotion words

Emotion words were extracted from the messages of the users self-identifying as having ASD in their conversations with Wysa. The emotion words are predicted by the Wysa AI models that include more than 200 words related to emotions from the user text input. Messages from users were then tagged when their text input contained one of the words (i.e., hopeless, scared, angry).

### Life challenges

Users selected the life challenges they wanted to address during the onboarding process in the Wysa app from a list of 16 possible life challenges. Life challenges were combined into the following clusters: stress and anxiety (anxiety, work stress, exam stress), energy and sleep (sleep, low energy, motivation), self-esteem (self-esteem, confidence), relationships (relationships, LGBTQ+), life events (pregnancy, health issues), low mood and depression (depression and loneliness), trauma and loss (trauma, loss).

## Data analysis

Two thematic analyses were conducted with the messages provided by the users. The first thematic analysis was done with the messages of the general users (sample 1), including the keywords (e.g., ASD, autism, Asperger), and aimed to identify common themes of conversations related to ASD. Messages that were labeled “unclear messages” or included “typos” were excluded from the analysis. The second thematic analysis was conducted with the messages of users that self-identified as having ASD (sample 2). The procedures for the thematic analyses followed the guidelines suggested by Braun and Clarke (44). The authors read the messages multiple times to familiarize themselves with the content. An initial list of preliminary codes were written, then researchers went through the data to verify the relevance of the potential themes. After consensus, a final set of major themes and subthemes were identified. The final list of themes and definitions were provided to independent and blind coders. Codes were assigned to each message, and the interrater reliability was calculated using Cohen's Kappa.

Emotion words were combined by valence (negative, positive, and ambivalent), and the negative valence words were split into four themes (depression, anger, anxiety, and other). Descriptive statistics were analyzed for the most frequent emotion words, themes, and valence and for the frequency of life challenges.

## Results

### Thematic analysis #1 of messages from general users related to ASD

There were three major themes identified. The most frequent one was “others having ASD,” followed by “ASD diagnosis,” and “seeking help.” The inter-rater reliability (IRR) was 0.98, 0.92, and 0.72, respectively, representing an almost perfect IRR (45). When the IRR was below strong (i.e.,: 0.80–0.90), researchers met to discuss the discrepancies and solved them by consensus. The major theme, “ASD diagnosis,” included four subthemes. Concerns about having ASD was the most frequent subtheme, followed by experiencing ASD symptoms, inquiring about ASD, and diagnosis in progress. See Table 1 for the complete list of themes, subthemes, definitions, examples, and frequencies.

**Table 1:** Messages of general users related to ASD.

Themes	Subthemes	Definition	Examples	Frequencies	Inter-rater Reliability
Others having ASD		Talking about other's diagnosis or ASD symptoms.	"he has autism and doesn't tell me how he feels" or "they bully him because he has autism"	343	0.98
ASD Diagnosis				275	0.92
	Concerns about having ASD	The user mentions that they may have autism or that they have symptoms of autism, that they are worried about being diagnosed with autism, or that they do not want to be diagnosed with autism.	"I think I am autistic" or "I show a lot of symptoms of autism" or "I don't want to be diagnosed with asd"	199	–
	Experiencing ASD symptoms	The user mentions experiencing symptoms of ASD.	"I'm having a bit of an autistic meltdown" or "loud noises; lots of ppl talking (autism thing)"	38	–
	Inquiring about ASD	The user asks Wysa if they have autism, if Wysa can diagnose them with autism, or inquires about their diagnosis.	"Do I have autism?" or "How do I know if I have autism"	22	–
	Diagnosis in process	The user mentions that they are in the process of being diagnosed with ASD.	"I am getting diagnosed with autism" or "well i'm about to go get a test which determines if i have autism or not"	18	–
Seeking help for ASD		The user inquiries about information, tools, or resources for individuals with autism or how Wysa can help them.	"do you have anything for autistic people to do?” or "i think i might have autism but im not diagnosed. what do i do?"	43	0.72a

### Thematic analysis #2 of messages of individuals that self-identify as having ASD

There were four major themes identified. The most frequent one was “ASD diagnosis or symptoms,” followed by “negative reaction from others,” “positive comments,” and “unclear.” The IRR was almost perfect for ASD diagnosis or symptoms, strong for negative reaction from others and positive comments, and weak for unclear (45). For the unclear messages, researchers met to discuss the discrepancies and solved them by consensus. The category “ASD diagnosis or symptoms” included four subthemes. Distress about ASD was the most frequent subtheme, followed by describing ASD, disclosing diagnosis, and self-aversion. The category “negative reactions from others” included four subthemes. Feeling misunderstood was the most frequent subtheme, followed by social problems, rejection of diagnosis by others, being bullied, and abuse. See Table 2 for the complete list of themes, subthemes, definitions, examples, and frequencies.

**Table 2:** Selected messages of individuals that self-identify as having ASD.

Themes	Subthemes	Definition	Examples	Frequencies	Inter-rater Reliability
ASD Diagnosis or Symptoms				184	0.94
	Distress about ASD	The user mentions feeling distressed over symptom(s) of their autism, expresses frustration, mentions not being able to do something due to their autism, or expresses general distress over their autism.	"my autism means i won't understand anything in college" or "i can't do any of that i have autism…"	86	–
	Describing ASD	The user mentions or describes a symptom related to their ASD diagnosis or what it is like to have ASD.	"…I'm autistic i can't visualize things in my head very well" or "…i'm autistic so i don't like going out a lot or visiting friends"	43	–
	Disclosing Diagnosis	The user only mentions their diagnosis. Note that all messages will include disclosing of ASD, this category will focus on those that only mention the diagnosis in their message.	"I have autism" or "i'm an autistic girl"	39	–
	Self-Aversion	The user mentions that they do not want to have ASD.	"I dont want to be autistic anymore" or "sometimes i just wish i was born normal, not autistic."	19	–
Negative Reaction from Others				143	0.85
	Feeling Misunderstood	The user mentions feeling misunderstood by people due to their ASD.	"my dad and i misunderstand each other a lot,…. he also doesn't really understand my autism…" or "my therapist doesn't understand my autism"	47	–
	Social Problems	The user mentions difficulty with making friends, that they do not have friends, or talks about being lonely or socially isolated.	"… I want friends… I don't fit in…" or "…i just wish it was easier for me to reach out and speak to others." or "everyone avoids me…"	42	–
	Rejection of Diagnosis by Others	The user mentions someone not rejecting them for or not accepting their ASD diagnosis, or mentions being afraid of rejection due to their diagnosis.	"half my family can't or won't accept that i'm autistic" or "i also got diagnosed autism but everyone treats me like it's fake"	24	–
	Being Bullied	The user mentions being bullied (by anyone- family or friends) or being made fun of due to having ASD.	"my confidence. it's low. people bully me at school for having autism" or "i've been been bullied or made fun of my whole life"	18	–
	Abuse	The user mentions being abused.	"I was emotionally abused…" or "they abuse me because im autistic"	14	–
Positive Comments		The user's message has a positive tone to it or is uplifting. They set a positive intention or mention acceptance of their autism.	"I intend to start understanding my add and autism more and love and better myself in that process" or "…he accepts me for what what i am even though i'm autistic"	22	0.87
Unclear		The user's message doesn't fit into any of the categories.	"idk but for my asd i am using a floppy chain"	9	0.52a

a Inter-rater reliability was weak, discrepancies among the themes were solved by consensus.

### Emotion words

**Figure 2:**
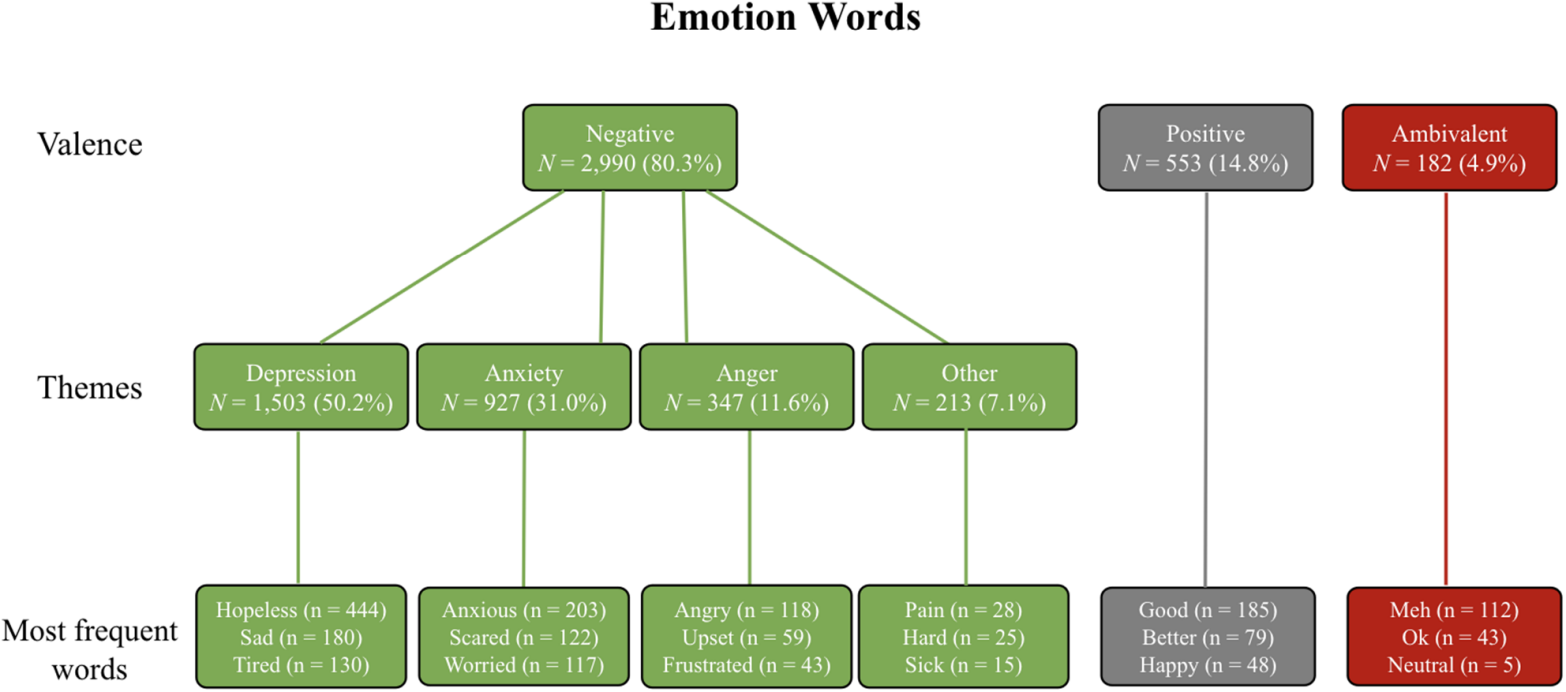
Frequency of emotion words by valance, themes, and single words.

There were a total of 3,725 emotion words mentioned in the messages of the individuals that self-identified as having ASD. The majority of those emotion words had a negative valence (80.3%), and few emotion words were positive (14.8%) or ambivalent (4.9%). Within the negative emotions, the most frequent theme was depression (50.2%), followed by anxiety (31%), anger (11.6%), and other (7.1%; e.g., ignored). Within the negative valence themes, for depression, the most frequent words were hopeless, sad, and tired; for anxiety, they were anxious, scared, and worried. For the positive valence words, the most frequently mentioned were good, better, and happy (See Figure 2).

### Life challenges

Of 232 users who identified as having ASD, 221 selected life challenges during the onboarding process. On average, users selected 8.21 life challenges (median = 8, range = 1–15). Energy and sleep was the most selected cluster of life challenges (*n* = 408, 22.8%), followed by stress and anxiety (*n* = 324, 18.1%), low mood and depression (*n* = 310, 17.3%), self-esteem (*n* = 308, 17.2%), relationships (*n* = 230, 12.9%), trauma and loss (*n* = 170, 7.8%), and life events (*n* = 70, 3.9%) (See Table 3).

**Table 3:** Clusters of life challenges selected*.*

	Life Challenges	*N*	%
1	Energy and Sleep	408	22.8
2	Stress and Anxiety	324	18.1
3	Low Mood and Depression	310	17.3
4	Self-Esteem	308	17.2
5	Relationships	230	12.9
6	Trauma and Loss	170	7.8
7	Life Events	70	3.9
	Total	1,820	100

## Discussion

Individuals with ASD face several obstacles to accessing treatment (5, 6). The advancement of technological resources, such as CA's, has provided an opportunity to increase access and help mitigate barriers to mental health resources (9, 14). However, there has been a limited number of studies utilizing CA's for individuals with ASD, a few studies on social skills (28–30), one on job interviewing skills (31), and one on ASD assessment (32). Most studies on CA's for individuals with ASD have small sample sizes and do not assess the themes of their conversations. Understanding the conversations that users have about ASD with a mental health CA can help tailor the needs of mental health chatbots toward the ASD population.

When analyzing the conversations related to ASD of the general users (thematic analysis #1), the most relevant themes were “others having ASD”, “ASD diagnosis”, and “seeking help for ASD”. “Others having ASD” was the most frequent topic, which addressed user's experiences with people in their lives with ASD. Some of these messages showed concern (e.g., “I'm really concerned about my autistic brother”) or empathy towards those with ASD (e.g., “everyone keeps talking bad about my friend that happens to have autism”), while other messages expressed the user's feelings about someone in their life with ASD (e.g., “my brother has autism and is right now very difficult to deal with. it is causing me and my mom a lot of stress”). This particular theme is expected as stress in relatives, as well as caregiver strain of individuals with ASD is well documented in research (46, 47). Another theme with considerable frequency was “ASD diagnosis,” where most users reported concerns about having ASD themselves or experiencing symptoms. A minor portion of users were explicitly seeking help (e.g., “I am struggling to deal with my son's autism diagnosis, how can you help?”) or asking for tools or resources (e.g., "do you have anything for autistic people to do"). These messages are consistent with the high stress experienced by individuals and families of those with ASD (46, 48, 49), and demonstrate how they share their emotions, and seek help from a mental health CA.

The second thematic analysis was conducted with 232 users that self-identified as having ASD and met the inclusion criteria. The most frequent theme was “ASD diagnosis or symptoms,” followed by “negative reaction from others,” and “positive comments.” The ASD diagnosis or symptoms theme included four subthemes. The most frequent subtheme was “distress about ASD” which reflected the burden that individuals with ASD experience (e.g., “i have autism that i don't understand fully yet”). Congruent with this subtheme, some users expressed aversion to having ASD (e.g., “I wish i wasn't autistic”). Altogether, these two subthemes highlight the need to develop chatbot resources to help alleviate the distress some individuals with ASD experience and, if appropriate, help them accept and perceive ASD in a more adaptive way. Another subtheme indicated that some users felt comfortable “disclosing their diagnosis” to a mental health CA. Given that previous studies have indicated that individuals with ASD are generally reluctant to disclose their diagnosis due to stigma and perceived negative outcomes (50), this finding highlights the potential benefit of chatbots as a mental health resource for the ASD community. Previous studies on chatbots for mental health have reported that some users felt that the chatbot would not judge them (26). Thus, it is possible that some users with ASD felt the CA would not judge them and experience less stigma associated with talking to a chatbot compared to a human.

“Negative reaction from others” was the second most frequent theme disclosed by individuals self-identified as having ASD. The negative reactions included “feeling misunderstood,” “social problems,” “rejection of diagnosis by others,” “being bullied,” and “abuse.” Overall, these negative reactions by the social environment of individuals with ASD have been documented in the literature. Individuals with ASD often have difficulties understanding those without ASD and vice versa, which is known as the double empathy problem (51). The double empathy problem underlines the communication barrier that may occur between those with ASD and those without, often making it difficult to connect through shared experiences or empathize with one another (51).

Additionally, individuals with ASD tend to be at a higher risk of being bullying victims than typically developing peers (52–54). Regarding abuse, those with ASD have been found to be more susceptible to abuse instances (55), 18.5% of children with ASD have been physically abused, and 16.6% have been sexually abused (56).

There were a few messages that expressed positive experiences within the context of ASD (e.g., “I have a good few musicians to relate to because, like myself, they are also autistic. this makes me feel proud to be me”). While these types of messages sound encouraging, they were the exception rather than the norm. Understanding the characteristics of these users with a positive attitude toward their condition may help inform strategies and understand protective factors for other users.

The emotion words of the users who self-identified as having ASD were also analyzed. Most emotion words had a negative valence, and few emotion words were positive or ambivalent. This is consistent with themes observed in the messages sent by individuals self-identifying as having ASD. The most frequent theme within the negative valence words was depression, followed by anxiety, anger, and other. Further, for depression, the most frequent words were hopeless, sad, and tired; for anxiety, they were anxious, scared, and worried. These findings are consistent with the struggles of individuals with ASD who experience increased emotional dysregulation (57), higher depression (58, 59), symptoms of anxiety (60), and lower positive well-being (61). Thus, future chatbot developments for individuals with ASD should include strategies for emotional regulation.

Similar to the findings of the message themes, there was a minor portion of positive valence words. The most frequently mentioned positive valence words were good, better, and happy. However, the positive valence words represent less than 15% of the emotion words reported. This can be explained by the higher rates of distress reported by individuals with ASD (62) and the type of conversations the chatbot yields. As with most therapeutic approaches, the chatbot, Wysa, focuses more on the problems that users are experiencing rather than asking about the positive aspects of their life. Although this is a common practice among therapists and chatbots, it may be important to incorporate more aspects from positive psychology to mental health chatbots to promote greater well-being in general and, in particular, for those with ASD.

Energy and sleep was the most frequently selected cluster of life challenges, followed by stress and anxiety, low mood and depression, self-esteem, relationships, trauma and loss, and life events. These findings are consistent with sleep difficulties commonly endorsed by individuals with ASD (63, 64). Additionally, these findings highlight the high comorbidity of anxiety and depression with ASD (65, 66). To note, individuals with ASD are more likely to experience depression and anxiety symptoms compared to typically developing individuals (67). Thus, CA's for individuals with ASD should include tools for coping with anxiety and depression.

It is important to note that most of the results reported in this study could have been impacted by specific features and designs of the Wysa app. For example, life challenges were selected from a specific list that Wysa provided to all users. Therefore, further research should be conducted to determine the generalizability of the current findings for the ASD population. Additionally, collaboration with individuals with ASD on developing an app to meet their specific needs could produce more generalizable findings for the ASD population.

## Limitations and future directions

The major limitation of the current study is the lack of demographic characteristics. While this is an important limitation, the lack of demographic information is due to the CA not collecting this data to protect users' confidentiality. Most digital therapeutics available in the market tend not to collect demographic information in order to ensure user privacy. However, this limits their ability to understand the population they serve and who benefits the most from their products. Researchers partnering with the industry should encourage their partners to collect demographic data, and future studies should include this data in their reports.

Another major limitation is that this study relied on users self-identifying as having ASD, and there was no external validation of the diagnosis. It is possible that some users who self-identified as meeting the criteria for ASD, in fact, did not hold or qualify for a diagnosis; therefore, the outcomes reported in this study should be used with caution. Future studies should include some level of external validation of the diagnosis. Additionally, as noted in prior studies (68), individuals who self-identify as having ASD while interacting with platforms such as chatbots may not be representative of the general population of individuals with ASD.

A third limitation of this study is the lack of context of the messages sent by users to the chatbot. When analyzing messages, the researchers did not have access to the full conversation exchanged between the user and the chatbot. Due to this, several messages were coded as unclear, even though they contained the keywords for this study. Future studies should consider the full conversational exchange between users and chatbots to allow for better understanding and context of the messages. Similarly, the emotion words were extracted from the messages without the context of the conversation. Thus, their interpretation should also be exercised with caution.

Finally, most of the data reported in the current study are qualitative. While qualitative data may help understand the needs of individuals with ASD, there is a need for quantitative data showing (a) how users that identify as having ASD utilize Wysa compared to individuals without ASD, and (b) assessing the efficacy of Wysa.

## Conclusions

The current study shows that users were open to disclosing their diagnosis, experiences, challenges, and emotions surrounding ASD, to a mental health chatbot designed for the general population. The most frequent conversations from general users were about others having ASD, ASD diagnosis, and seeking help for ASD. Furthermore, the conversations of those that self-identified as having ASD refer to their diagnosis, negative reactions from others, and a few positive comments about ASD. The majority of the emotion words were related to negative emotions, such as depression, anxiety, and anger. Overall, these themes highlight the potential of mental health CA's to discuss ASD-related topics with their users and become a source of help. Thus, there is a need to create mental health chatbots that are tailored to meet the unique needs of those with ASD.

## Data Availability

The data analyzed in this study is subject to the following licenses/restrictions: Wysa prioritizes security and privacy of its users; therefore, authors were provided with limited and de-identified data that is unable to be made publicly available. Requests to access these datasets should be directed to Dr. Eduardo Bunge, ebunge@paloaltou.edu.
